# Optimising the mutation screening strategy in Marfan syndrome and identifying genotypes with more severe aortic involvement

**DOI:** 10.1186/s13023-020-01569-4

**Published:** 2020-10-15

**Authors:** Roland Stengl, András Bors, Bence Ágg, Miklós Pólos, Gabor Matyas, Mária Judit Molnár, Bálint Fekete, Dóra Csabán, Hajnalka Andrikovics, Béla Merkely, Tamás Radovits, Zoltán Szabolcs, Kálmán Benke

**Affiliations:** 1 Heart and Vascular Center, Semmelweis University, Városmajor u. 68, Budapest, 1122 Hungary; 2 Hungarian Marfan Foundation, Városmajor u. 68, Budapest, 1122 Hungary; 3 Laboratory of Molecular Genetics, Central Hospital of Southern Pest, National Institute of Hematology and Infectious Diseases, Albert Flórián út 5-7, Budapest, 1097 Hungary; 4 Department of Pharmacology and Pharmacotherapy, Semmelweis University, Üllői út 26, Budapest, 1085 Hungary; 5 Center for Cardiovascular Genetics and Gene Diagnostics, Foundation for People With Rare Diseases, Wagistrasse 25, 8952 Schlieren, Zurich, Switzerland; 6 Institute of Genomic Medicine and Rare Disorders, Semmelweis University, Tömő u. 25-29, Budapest, 1083 Hungary

**Keywords:** Marfan syndrome, Genetic testing, Aortic involvement, Risk stratification, FBN1, Next-generation sequencing, MLPA, Gene panel, Cardiac surgery

## Abstract

**Background:**

Marfan syndrome (MFS) is a systemic connective tissue disorder with life-threatening manifestations affecting the ascending aorta. MFS is caused by dominant negative (DN) and haploinsufficient (HI) mutations of the *FBN1* gene. Our aim was to identify mutations of MFS patients with high detection rate and to investigate the use of a gene panel for patients with Marfanoid habitus. We also aimed to examine correlations between genotype and cardiovascular manifestations to predict “malignant” mutations.

**Methods:**

136 individuals were enrolled. In the first phase, next-generation sequencing (NGS) and Sanger sequencing were performed for 57 patients to screen the *FBN1* gene, followed by multiplex ligation-dependent probe amplification (MLPA) in negative cases. For repeated negative results, NGS gene panel involving 9 genes was used. In the second phase, 79 patients were tested primarily with the same gene panel, negative samples were tested by MLPA.

**Results:**

84 pathogenic mutations were detected, out of which 78 affected *FBN1*, 6 non-*FBN1* mutations (2 *TGFB2*, 1 *TGFBR2*, 2 *TGFBR1*, 1 *SMAD3*) are associated with Loeys-Dietz syndrome (LDS). LDS patients had lower systemic score and they were younger, but their aortic involvement did not differ. MLPA detected 4 multi-exon deletions of *FBN1* gene, which could not be identified by our first-step screening method. Aortic involvement (aortic dissection and/or dilation) did not differ significantly among HI and DN mutations (*p* = 0.061). Combined group of HI and DN mutations eliminating a disulphide-bonding cysteine (DN Cys) had significantly higher aortic involvement rate than DN mutations not eliminating a disulphide-bonding cysteine (DN non-Cys) (*p* < 0.001). Patients with DN Cys required significantly more aortic surgeries than HI and DN non-Cys mutations (*p* = 0.042 and *p* = 0.015, respectively).

**Conclusions:**

Due to the relevant number of mutations affecting genes other than *FBN1*, preferred approach for testing individuals with Marfanoid habitus is using a gene panel rather than single-gene analysis, followed by MLPA for negative samples. DN Cys and HI mutations should be considered as risk factors for aortic involvement. Genetic testing for patients with Marfanoid features and a systemic score under 7 is recommended, as LDS patients may have lower scores, but they may have severe cardiovascular manifestations.

## Background

Marfan syndrome (MFS) is a systemic connective tissue disorder with a prevalence of 1:3000–1:5000 [1]. The main clinical manifestations typically involve the cardiovascular (CV), musculoskeletal and ocular systems [2].

The disease is inherited in an autosomal dominant manner and caused by mutations of the *FBN1* gene, which is located on the long arm of chromosome 15 (15q21.1) and consists of 65 coding exons [3]. It encodes the fibrillin-1 protein, which is secreted into the extracellular matrix and cooperates with elastin to build up the connective tissue through the formation of elastic fibres. It also has structural roles even independently from elastin, for example building up ciliary zonules in the eye [4]. Its regulatory function is to keep transforming growth factor-β (TGF-β) in an inactive form [5]. A particularly important amino acid in the structure of fibrillin-1 is cysteine, of which more than 360 can be found in the protein. Cysteine plays a critical role in the stability of fibrillin-1 due to the formation of disulphide bridges [6]. Mutations that eliminate this amino acid have been proved to result in more severe CV involvement than the ones introducing new cysteine [7, 8], emphasising the particular role of this disulphide-bonding amino acid.

To date, more than 3000 genetic variants of the *FBN1* gene have been reported in the Human Gene Mutation Database [9]. They spread throughout the gene, affecting all exons [3]. Around half of them are missense mutations, the others are nonsense, splice-site mutations, and small in-frame or, frameshift insertions and deletions (indels, ≤ 50 bp) or copy number variations (CNVs, > 50 bp) [9, 10]. CNVs are deletions and duplications affecting more than 50 bp and they account for around 10% of disease-causing genetic variants in Mendelian diseases [11]. *FBN1* mutations can be classified into haploinsufficient (HI) and dominant negative (DN) groups based on their effect on the encoded protein. HI mutations result in the reduction of protein quantity, therefore in this case, only/mainly the normal protein can be found in the connective tissue [12, 13]. As opposed to that, DN mutations lead to abnormal protein structure, so the connective tissue contains both the normal and abnormal fibrillin-1 [14].

Genetic testing of the *FBN1* gene has been receiving growing attention in the past few years and besides clinical features, it has become one of the key criteria of the diagnosis of MFS in the revised Ghent nosology [2].

To date, only a few well-established connections between genetic background and phenotype have been described [15], e.g. *FBN1* mutations affecting a cysteine amino acid are more likely to lead to ectopia lentis [16]. There is also a strong relationship between the severity of MFS and a specific part of the gene: if the so-called neonatal region, which is spread throughout the exons 24–32, is affected, then there is a significantly increased chance for the occurrence of neonatal MFS, which is the most severe form of the disease [16]. Mutations in this region were found to lead to a higher probability of ascending aortic dilation, aortic surgery, mitral valve abnormalities, ectopia lentis, scoliosis and shorter survival even when neonatal MFS was excluded. Therefore, a genetic variant in the region of exons 24–32 can result in a more severe phenotype and can be an indicator of early onset aortic risk even in the absence of neonatal MFS [16].

Establishing the differential diagnosis is an important aspect as many related disorders of MFS lead to similar clinical appearance, but require different therapeutic approach [2]. Comparing to MFS, the Marfan-related Loeys-Dietz syndrome (LDS) may present with more aggressive clinical presentation, characterised by rapidly growing aneurysms, and aortic dissections occurring at a younger age and with smaller diameters. Furthermore, LDS dissections can take place in the peripheral arteries too. These indicate the need for more frequent and extended surveillance and lower aortic diameter as the indication criterion for a prophylactic surgery [17]. Therefore, differentiating between MFS and LDS carries huge clinical importance. In LDS, the *TGFBR1*, *TGFBR2*, *SMAD3*, *TGFB2*, *TGFB3* and *SMAD2* genes are affected [18]. On the other hand, some of the diseases that result in a Marfan-like appearance may not lead to severe CV complications. One example of that is congenital contractural arachnodactyly (Beals syndrome), which is caused by a mutation in the *FBN2* gene [19]. Therefore, it is pivotal to identify the actual syndrome the patient has, so the proper management can be carried out.

Some of the characteristic features of MFS are aortic dilation and dissection, mitral valve prolapse, chest deformities, dolichostenomelia, scoliosis, skin striae, myopia and ectopia lentis [2].

The most dangerous, life-threatening complication of MFS and other genetic aortopathies is aortic dissection, which occurs at the average age of 63 years in the general population, ~ 38 years in MFS [20] and ~ 27 years in LDS patients [21]. Approximately two-thirds of aortic dissections belong to group type A in the Stanford classification system, meaning that they involve the ascending aorta. Without surgical intervention, an acute type A aortic dissection has a mortality rate of 20% after 24 h and it increases to 30% after 48 h [22]. The mortality of the operation of an acute type A aortic dissection can reach even 20%, while it is only around 1.5% in case of prophylactic surgery [23].

The indication for prophylactic surgery is based on aortic diameter. The threshold is 50 mm for MFS patients, which goes down to 45 mm in the presence of any of the risk factors stated in the European Society of Cardiology (ESC) guidelines [24]. The main concern is that a relevant number of aortic dissections occur at smaller diameters. In a study by Neri and his colleagues, one-third of aortic dissections took place at normal or mildly enlarged diameters [25], and in another article, Kim and his colleagues reported a smaller than 45 mm aortic diameter for 26% of dissections [26]. However, too early, and unnecessary operations should be also avoided. As aortic diameter alone is not appropriate to predict aortic dissection, a model that could precisely determine the risk and the possible onset should be created [27–29].

Therefore, our research aimed to identify the pathogenic genetic variants of clinically diagnosed MFS patients with the highest achievable detection rate. We also aimed to examine the relevance of the use of a multi-gene panel in the investigation of patients with a Marfanoid habitus.

Our further objective was to identify genotype–phenotype correlations that could improve the risk stratification for severe aortic events and could be applied within clinical settings.

## Patients and methods

### Studied population

At the Heart and Vascular Center of Semmelweis University in Budapest, Hungary, the Marfan out-patient clinic was established to follow-up and treat patients and also help to orientate people with the clinical suspicion of the disease. MFS patients are registered in the Hungarian Marfan Register, maintained by the Hungarian Marfan Foundation. The database includes more than 500 patients [30].

Until December 2019, 136 patients underwent genetic testing. After informed genetic counselling and written consent, blood sample was collected, and DNA was isolated (ETT TUKEB 12751-3/2017/EKU).

### Study design

The study was divided into two distinct phases. In the first phase, 57 patients were involved. The second phase included patients with negative results from the first phase and further 79 newly enrolled patients. The participants enrolled at different periods are referred to as first (n = 57) and second (n = 79) set of patients. The inclusion criterion in the first phase was the clinical diagnosis of MFS; in the second phase, patients with the clinical diagnosis of MFS and patients with Marfanoid habitus were included. We defined Marfanoid habitus as having a systemic score of at least 5 points. The clinical diagnosis was based on the revised Ghent nosology [2].

The first phase took place in 2017 and 2018, while the second phase was carried out in 2019. The reasons for establishing two distinct phases were the questions emerging during the experiment, which required more advanced diagnostic tools. Patients were selected in the order of visiting the clinic.

We decided to include first-degree relatives who presented with Marfanoid features regardless of their systemic score, as the disease shows a high interpersonal phenotypic variability. The 136 patients, including 18 first-degree relatives, came from 118 families.

### Single-gene analysis

- Step 1: In the first phase, we screened for mutations of the *FBN1* gene with the use of next-generation sequencing (NGS) technique as previously described [31]. We applied a Roche GS Junior platform.
- Step 2: Homopolymer regions were investigated with Sanger sequencing with the use of ABI Prism 310 Genetic Analyser (Applied Biosystems) and all the detected (likely) pathogenic mutations were confirmed by this technique.

### Multi-gene panel analysis

In the second phase of the study, we applied an NGS based multi-gene panel, which covered the potential genes of Marfan syndrome and its overlapping related disorders, to increase our detection rate and to enable a differential diagnosis to be established. These involved the following 9 genes: *ACTA2, COL3A1, FBN1, KCNN1, MYH11, SMAD3, TGFB2, TGFBR1* and *TGFBR2*. The mutations of these genes can lead to heritable aortopathies, including MFS, LDS, vascular Ehlers-Danlos syndrome (vEDS) and familial thoracic aortic aneurysm and dissection (FTAAD) [32]. With the multi-gene panel method, we examined the samples of a total number of 96 patients. The genomic DNA libraries were prepared by using QIAseq targeted DNA custom panel (QIAGEN, USA); for the subsequent NGS, we applied the Illumina MiSeq platform (Illumina, San Diego, USA). The Variant Call Format (VCF) files were annotated with the SnpEff software [33] and the ClinVar database [34]. The variant classification was carried out by using the VariantAnalyzer software developed by the Budapest University of Technology and Economics. Various databases were used to interpret the pathogenicity of a rare variant. These included Varsome [35], Human Gene Mutation Database [9], Universal Mutation Database [36, 37], dbSNP [38], and gnomADv2.1 non-Finnish population. The variant classification was performed according to ACMG guidelines [39].

Pathogenic and likely pathogenic missense mutations were classified as DN variants, while nonsense, splice-site and frameshift mutations and CNVs were considered as HI genetic variants.

### MLPA

When no pathogenic mutations were detected by sequencing, we applied the multiplex ligation-dependent probe amplification (MLPA) technique to screen for CNVs of the *FBN1* and *TGFBR2* genes [31] (MRCHolland, Amsterdam, the Netherlands), as the sequencing methods used in this study are not or less capable to detect CNVs in heterozygous form. MLPA was performed in 19 and 30 patients in phase 1 and 2, respectively.

### Investigation of genotype–phenotype correlations

When (likely) pathogenic genetic variants were identified, we examined the correlations between the severity of CV manifestations and the genotype. We focused on the involvement of the ascending aorta, including dilation and dissection. Dilation is defined by the Z-score reaching and exceeding 2 above 20 years, and 3 below 20 years [2]. The frequency of these CV complications were compared between (likely) pathogenic variant positive and negative patients and also between patients with HI and DN mutations of the *FBN1* gene. Based on the crucial role of cysteine in protein structure, we further stratified DN mutations into genetic variants that resulted in the elimination of disulphide-bonding cysteine (DN Cys) and the ones that did not substitute such amino acid (DN non-Cys). We analysed the need for aortic surgeries among the different mutation types of the *FBN1* gene. We also compared the aortic involvement of MFS/LDS patients to the group of no identified mutations. Finally, we investigated the well-known genotype–phenotype correlations in our patient cohort.

### Statistical analysis

We used two-sample *t*-test and chi-squared test to compare certain groups; results were considered significant at *p* < 0.05. To describe the general characteristics of the examined population, we calculated the mean and 95% confidence interval; for the systemic score, we used median with first and third interquartile range.

## Results

**Fig. 1:**
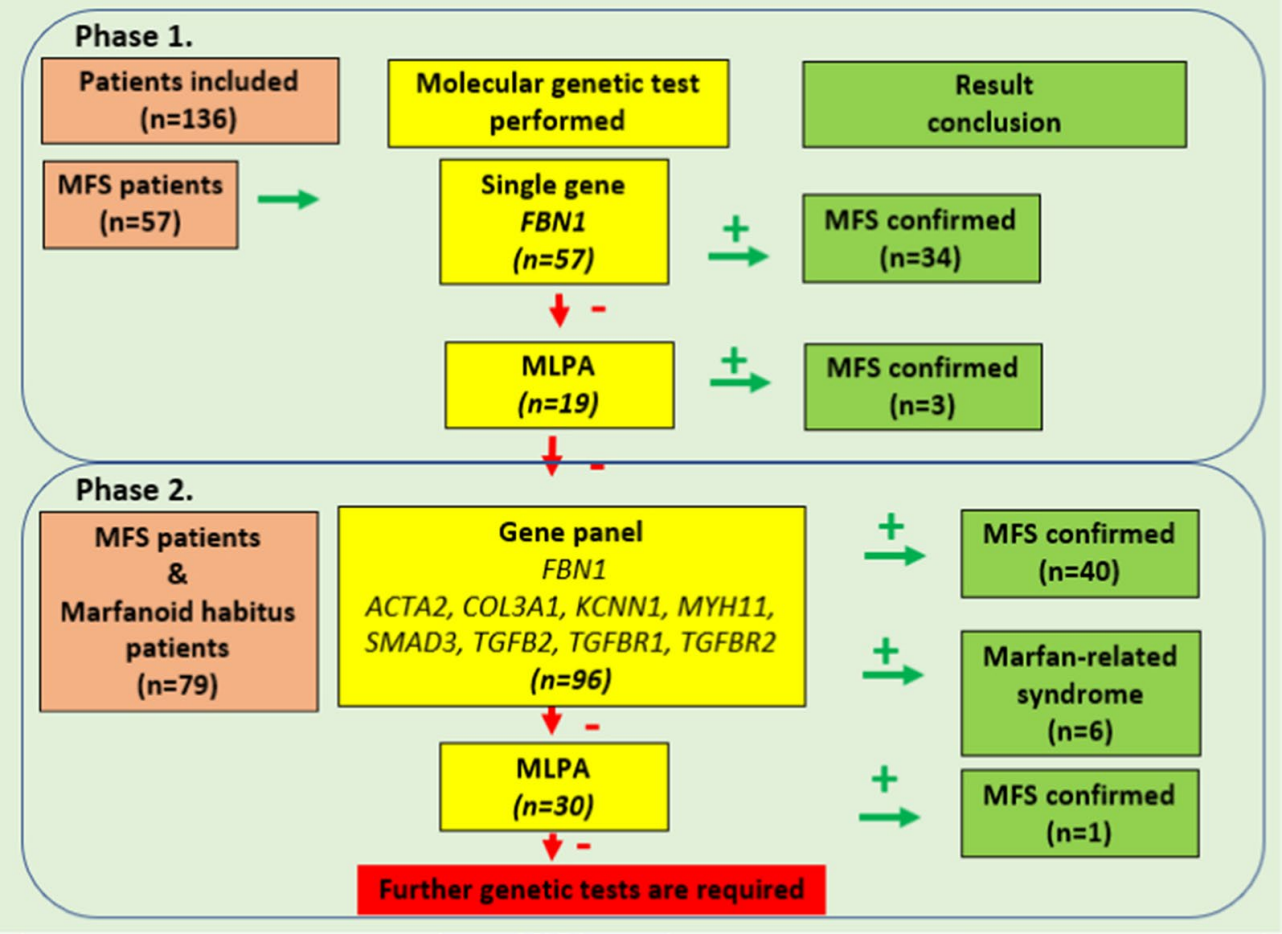
Summary of the genetic testing steps. Genetic screening steps for two sets of patients. We started the genetic testing with sequencing the *FBN1* gene. The negative samples were further investigated with MLPA technique. We applied an NGS gene panel for the samples with repeated negative results. We applied the gene panel followed by MLPA for the second set of patients. The two phases of the study are indicated

Figure 1 shows the distribution of genetic screening steps applied for the two sets of patients and the results of the two phases of the study.

### Results of phase I

The examined population in the first phase of the genetic testing consisted of 19 men (33%) and 38 women (67%) with an average age of 33 (30–37) years at the time of the genetic screening. Their median systemic score was 8 (ranging from 7 to 10).

Altogether 34 (likely) pathogenic mutations of the *FBN1* gene were identified with the use of NGS and Sanger sequencing, resulting in a detection rate of 60% (34/57). These included 17 missense (50%), 8 nonsense (23%), 5 frameshift (15%) and 4 splice-site (12%) mutations. We identified 2 missense variants of unknown significance (VUS). In 19 of the 21 MFS cases with no identified *FBN1* mutations, we applied MLPA to screen for CNVs and we had three positive results in the *FBN1* gene (16%). In one patient, it caused the deletion of exons 1–2 and for the other patients, exons 2–4 were deleted.

The average age of patients with identified mutations was 37 (32–41) years with a median systemic score of 8 (ranging from 7 to 10).

The 20 MFS patients with no detected (likely) pathogenic *FBN1* mutation had an average age of 27 (22–32) years and a median systemic score of 7.5 (ranging from 6 to 9). Therefore, in terms of the systemic score, there was no significant difference between people with or without identified mutations (*p* = 0.100), but people without identified mutations were significantly younger (*p* = 0.011).

### Results of phase II

In the second phase of the research, 30 pathogenic variants were identified, 27 of which affected the *FBN1* gene (90%), including 3 missense (11%), 7 nonsense (26%), 7 frameshift (26%) and 10 splice-site (37%) mutations. One nonsense and 2 frameshift mutations affected *TGFBR2* (3.3%) and *TGFB2* (6.7%), respectively. Also, 16 likely pathogenic mutations were detected, 13 of which affected the *FBN1* gene (81%), including 10 missense mutations (76.9%), and 3 in-frame deletions (23.1%). Two likely pathogenic missense variants affected the *TGFBR1* gene (12.5%). One likely pathogenic missense variant was identified in the *SMAD3* gene. The average age of the group of people with pathogenic or likely pathogenic genetic variants was 36 (33–40) years and their median systemic score was 8 (ranging from 7 to 9).

**Fig. 2:**
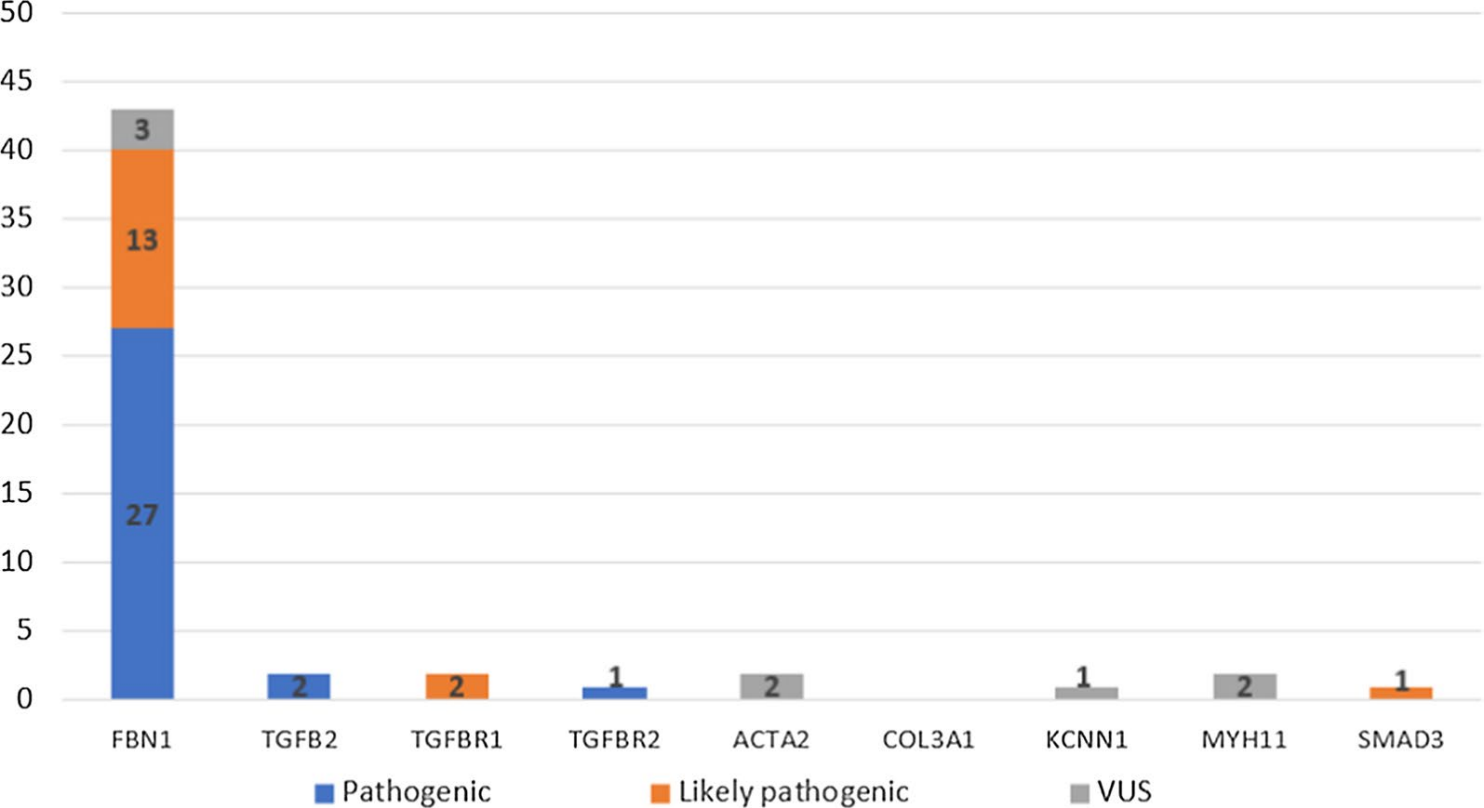
Results of the gene panel. The diagram shows the identified pathogenic, likely pathogenic mutations and the variants of unknown significance

In 8 samples, we detected variants of unknown significance (VUS). One missense and 2 non-coding VUS affected the *FBN1* gene (37.5%) and 2 missense VUS were found in *MYH11* (25%), 2 VUS in *ACTA2* (25%), and 1 VUS in the *KCNN1* (12.5%) gene. Four of them were detected in people with an identified (likely) pathogenic variant, and the other 4 appeared to be the only detected mutation. The latter 4 genetic variants need to undergo further investigations. The distribution of the mutations identified by the gene panel can be seen in Fig. 2.

From the second set of patients, 30 variant negative samples were further analysed using MLPA (Fig. 1). One CNV was identified, which involved the deletion of the exons 3–4 of the *FBN1* gene.

### Overall results

In the first set of patients, the detection rate of the *FBN1* genetic variants appeared to be 65% (37/57), while in the second set of patients the *FBN1* mutation identification rate was 52% (41/79) (*p* = 0.13). Overall detection rate of the *FBN1* mutations was 57% (78/136).

Altogether 84 positive mutations of the 136 examined patients were identified, which is a 62% overall success rate in mutation detection. Results were considered positive in the case of pathogenic and likely pathogenic variants, VUS and the rest accounted for the negative results [32]. The general characteristics and detailed Ghent nosology features of the patients with and without a positive mutation can be seen in Tables 1 and 2. Apart from their Body Mass Index, the general characteristics of the two population did not differ. The identified pathogenic and likely pathogenic genetic variants can be found in the additional file (see Additional file 1).

**Table 1:** General characteristics I

	Studied population (n = 136)	Positive for mutation (n = 84)	Not positive for mutation (n = 52)	*p* value (positive vs. not positive)
Male (%)	46	42	54	0.166
Age	35 (33–38)	37 (34–40)	33 (28–37)	0.113
Anthropometric (measured)				
Height (cm)	183.7 (181.8–185.7	183.4 (180.9–185.8)	184.4 (181.2–187.6)	0.604
Lower segment (cm)	96.1 (94.6–97.5)	96.3 (94.4–98.1)	95.8 (93.3–98.3)	0.748
Arm span (cm)	188.1 (186.0–190.3)	188.4 (185.6–191.1)	187.8 (184.2–191.3)	0.786
Footsize	42.8 (42.3–43.4)	42.8 (42.2–43.5)	42.9 (42.0–43.8)	0.956
Weight (kg)	70.3 (67.3–73.3)	72.0 (68.3–75.7)	67.5 (62.3–72.7)	0.153
Anthropometric (calculated)				
Body Mass Index (BMI; kg/m^2^)	20.7 (20.0–21.4)	21.3 (20.4–22.1)	19.7 (18.5–21.0)	*0.042*
Body surface area (m^2^)	1.88 (1.84–1.93)	1.91 (1.85–1.96)	1.85 (1.77–1.93)	0.230
Upper segment–lower segment ratio (USLS)	0.92 (0.90–0.94)	0.91 (0.88–0.93)	0.93 (0.90–0.97)	0.218
Arm span-height ratio (ASHR)	1.024 (1.018–1.030)	1.027 (1.019–1.036)	1.018 (1.008–1.028)	0.158
Systemic score	8 (7–9)	8 (7–9)	8 (7–9)	0.249

The table shows the general characteristics of the examined cohort with and without identified (likely) pathogenic genetic variants

The italics emphasises that the result is significant

**Table 2:** Ghent nosology

	Examined population (n = 136)	Mutation identified (n = 84)	No mutation identified (n = 52)
Ghent nosology (%)			
Mitral valve prolapse	66	74	54
Dilation or dissection of descending aorta	4	5	4
Pectus carinatum	43	45	40
Pectus excavatum requiring surgery	9	12	4
Reduced upper to lower segment ratio	19	21	15
Increased arm span to height ratio	23	27	15
Wrist sign	81	82	79
Thumb sign	85	92	73
Scoliosis of > 20° or spondylolisthesis	68	74	60
Severe scoliosis	29	36	17
Reduced extension at the elbows	6	10	0
Medial displacement of the medial malleolus causing pes planus	46	51	37
Heel deformity	20	19	21
Protrusion acetabulae	0.7	1	0
Pectus excavatum of moderate severity	22	26	15
Asymetric chest	46	50	40
Joint hypermobility	47	57	31
Highly arched palate with crowding of teeth	60	65	50
Dolichocephaly	21	24	15
Enophtalmos	14	15	12
Downslanting palpebral fissure	28	31	23
Malar hypoplasia	13	17	6
Retrognathia	40	48	29
Ectopia lentis	21	32	4
Myopia over 3 diopter	40	45	31
Abnormally flat cornea	0.7	1	0
Increased axial length of globe	2	4	0
Hypoplastic iris	1.5	2	0
Spontaneous pneumothorax	7	5	10
Apical blebs, bullae	1.5	2	0
Lumbosacral dural ectasia	3	2	4
Striae atrophicae (stretch marks)	65	65	65

This table shows the Ghent nosology features of the examined cohort with and without an identified (likely) pathogenic sequence variant

Out of the positive variants, 6 affected genes other than *FBN1* (7%); these 6 patients received the diagnosis of LDS. The general characteristics of MFS and LDS patients are shown in Table 3. The mutations affecting the *FBN1* gene led to a significantly higher systemic score than the ones found in other genes (*p* = 0.013). However, there was a tendency in the non-*FBN1* group to be younger (*p* = 0.057). Despite this, all the patients with a non-*FBN1* mutation had a dilated ascending aorta, and 2 of them had already undergone prophylactic aortic root surgeries.

**Table 3:** General characteristics II

	MFS (n = 78)	LDS (n = 6)	*p* value
Male (%)	41	50	0.667
Age	37.5 (34.4–40.6)	26.7 (19.8–33.5)	0.057
Anthropometric (measured)			
Height (cm)	183.5 (180.9–186.0)	182.2 (170.2–194.2)	0.777
Lower segment (cm)	96.7 (94.8–98.5)	91.6 (82.2–101.0)	0.153
Arm span (cm)	189.0 (186.1–191.8)	181.5 (168.2–194.8)	0.138
Foot size	42.9 (42.2–43.6)	42.6 (37.9–47.3)	0.854
Weight (kg)	72.7 (68.9–76.6)	63.3 (48.8–77.9)	0.164
Anthropometric (calculated)			
Body Mass Index (BMI; kg/m^2^)	21.49 (20.59–22.39)	18.94 (15.97–21.93)	0.106
Body surface area (mt)	1.92 (1.86–1.98)	1.78 (1.53–2.03)	0.209
Upper segment–lower segment ratio (USLS)	0.90 (0.88–0.92)	1.01 (0.84–1.18)	*0.02*
Arm span-height ratio (ASHR)	1.030 (1.021–1.039)	0.997 (0.980–1.012)	*0.025*
Systemic score	8 (7–9)	6.5 (6–7)	*0.013*

General characteristics of MFS and LDS patients are shown

The italics emphasises that the result is significant

### Genotype–phenotype correlations

**Fig. 3:**
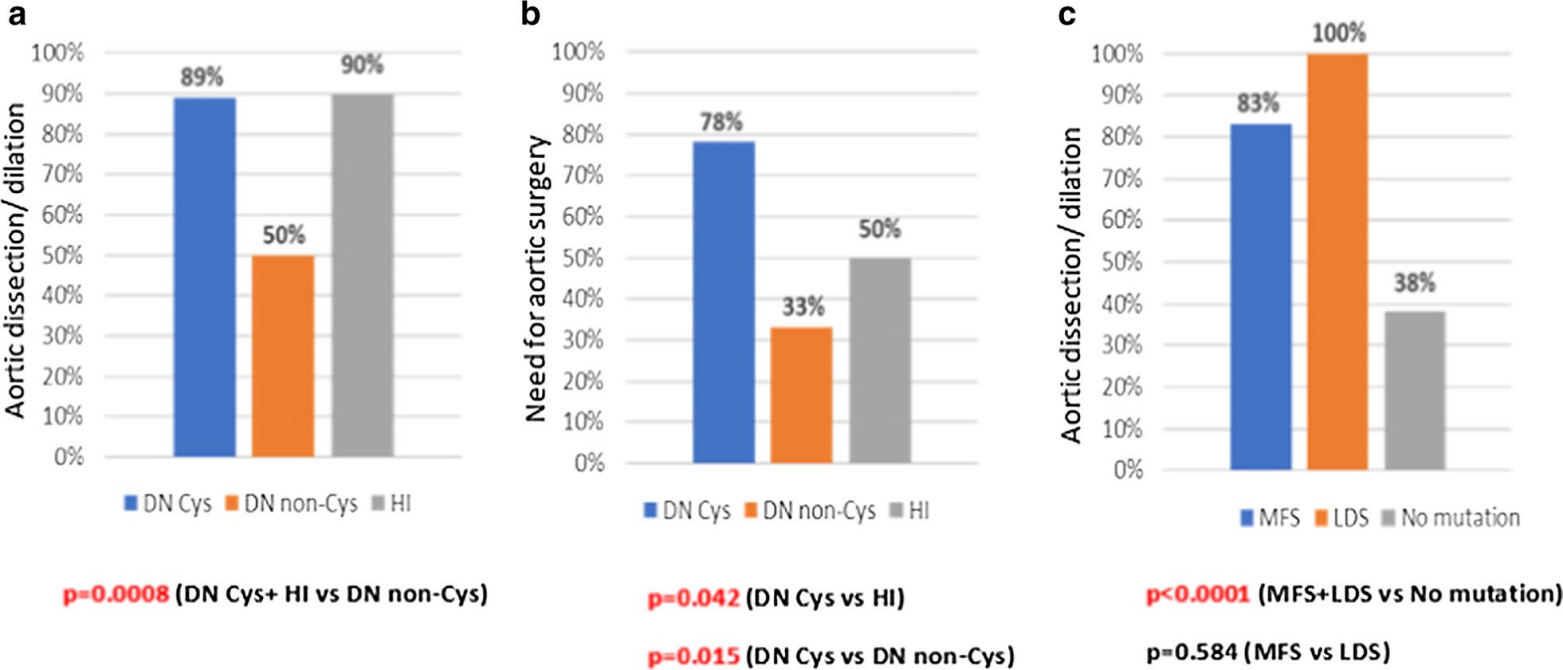
Genotype–phenotype correlations. **a** The combined group of HI and DN Cys led to aortic involvement significantly more frequently than DN non-Cys genetic variants. **b** The need for aortic surgery was significantly more common for DN Cys mutations than for the other two types. **c** Individuals with detected mutations showed a significantly higher aortic involvement rate than individuals without an identified (likely) pathogenic variant. No difference could be observed between MFS and LDS patients

We investigated the correlations between the genetic background and the clinical manifestations of our patients. First, we examined the aortic involvement (dissection and/or dilation) among the DN and HI groups of the *FBN1* gene: 73% (22/30) of individuals with DN and 90% (43/48) of individuals with a HI genetic variant showed aortic involvement (*p* = 0.061)*.* Then we investigated DN Cys and DN non-Cys variants, 89% (16/18) of DN Cys mutations led to aortic dilation and/or dissection, while aortic involvement was only 50% (6/12) in case of DN non-Cys variants (*p* = 0.018). To make a classification which could be useful within clinical settings, we placed the DN Cys and HI mutations into one group and compared it to the DN non-Cys mutations. According to our results, DN non-Cys led to aortic involvement significantly less frequently than the combined group (*p* < 0.001) (Fig. 3a)*.* DN Cys genetic variants required aortic surgeries significantly more frequently than HI ones (78% vs 50%; *p* = 0.042) and the DN non-Cys mutations (78% vs 33%; *p* = 0.015) (Fig. 3b). The mean age at the time of surgery was 36 (28–44) years for DN Cys-, 32 (15–48) years for DN non-Cys- and 35 (31–38) years for HI patients. No significant difference could be observed in the age at the time of surgery among the 3 mutation types (DN Cys vs DN non-Cys *p* = 0.605; DN non-Cys vs HI *p* = 0.524; DN Cys vs HI *p* = 0.757).

The mean age at the last follow-up did not differ among the 3 mutation types. It was 43 (36–51) years in the DN Cys-, 34 (28–40) years in the DN non-Cys- and 38 (34–41) years in the HI group (DN Cys vs DN non-Cys *p* = 0.074; DN Cys vs HI *p* = 0.151; DN non-Cys vs HI *p* = 0.382).

All the individuals with the diagnosis of LDS (6/6) had either aortic dissection or dilation; aortic involvement was 83% (65/78) for MFS patients with identified genetic background and 38% (20/52) for individuals without a detected sequence variant. Therefore, people with identified mutations had aortic involvement significantly more frequently than the patients without a detected mutation (*p* < 0.001). No difference could be observed between the MFS and LDS groups (*p* = 0.584) (Fig. 3c). The general characteristics of MFS and LDS patients can be found in Table 3, showing that LDS patients have less severe anthropometric manifestations compared to MFS-cases.

Interestingly, 8 of the (likely) pathogenic *FBN1* variants occurred in the neonatal region (exons 24–32) and only one of them led to severe CV manifestation (prophylactic aortic surgery at the age of 17). The other cases presented as classical MFS. Of the 7 cases with a mutation in the neonatal region that presented as classical MFS, 6 developed aortic dilation without reaching the threshold for prophylactic surgery. There was no significant difference in terms of aortic involvement between patients with a mutation in exons 24–32 (7/8) and patients with a mutation not affecting exons 24–32 (58/70) (*p* = 0.739).

In our patient cohort, 61% of DN Cys, 27% of HI and 14% of DN non-Cys mutations resulted in the dislocation of the lens. As a result, DN Cys led to ectopia lentis significantly more frequently than the HI, DN non-Cys and the combined group of HI and DN non-Cys mutations (*p* = 0.011, *p* = 0.008 and *p* = 0.003, respectively). This finding is consistent with the literature [16, 40].

## Discussion

Currently, the most widely applied algorithm for genetic testing in MFS patients is the sequence analysis of the *FBN1* gene followed by CNV screening, and in negative cases the investigation of the *TGFBR1* and *TGFBR2* genes [41].

In our two-phase genetic testing study, we included 136 patients with either a clinical diagnosis of MFS or Marfanoid habitus, the first thorough genetic screening study of the Hungarian Marfan population. Due to the overlapping features of MFS and its related disorders, we used a multi-gene panel to investigate the relevant genes.

The mutation detection rate of 62% in our study is in the range found in the literature. The success of *FBN1* mutation identification can be as high as 93% in individuals with clinically diagnosed MFS. However, this rate shows a huge variability. Arnaud and his colleagues had a 56% detection rate in their study [42] while Baetens reached a 92% success rate [31]. The previously mentioned studies only investigated the *FBN1* gene, but examples of multigene testing are also available. Yang and his colleagues used a 15-gene aortopathy panel for 248 patients and they identified 92 (37.1%) (likely) pathogenic mutations, 89% of them affecting the *FBN1* gene [43]. In the study of Lerner-Ellis, 594 individuals with the suspicion of Marfan syndrome, Loeys-Dietz syndrome and Thoracic Aortic Aneurysms and Dissections were tested, and 112 individuals had a positive result (19%). The authors state that the reason behind the low detection rate is the errors made during the patient selection process as they were not referred for testing by individuals with expertise in the field [44]. In a study by Wooderchak-Donahue, a 10-gene panel was applied, and 10.3% of the 175 tested individuals appeared to be positive, while 18.3% had a VUS [41].

When we compared the general traits of patients with a detected mutation to the individuals without a detected mutation, only their Body Mass Index (BMI) differed significantly, the characteristic Marfanoid features and their systemic score were similar. This could suggest that errors in our patient selection were not the main reason for not reaching the maximal detection rate.

We investigated 9 genes, so one explanation for the lower success rate can be the involvement of other genes that were not covered by our gene panel. Mutations in the intronic regions have been reported [33], which could also contribute to our number, as we have not sequenced deep/non-canonical intronic regions.

We have identified 4 CNVs of the *FBN1* gene by MLPA, and they accounted for ~ 5% of the detected mutations. Altogether 49 MLPA tests were carried out, so the detection rate was ~ 8%. Without this technique, these genetic variants would not have been detected, so the patients could not have received the benefits provided by an identified mutation.

Yang and his colleagues applied MLPA for 115 samples that came back negative after a 15-gene panel. As a result, they found 5 large deletions in the *FBN1* gene (4.3%) [45]. Consistently with our previous study, these findings highlight the importance of CNV screening in point-mutation negative cases, to increase the detection rate of disease-causing genetic variants [11].

Increasing the mutation detection rate is highly important as identifying the patients’ pathogenic mutation carries several benefits.

First, having an identified mutation helps to confirm the diagnosis of the disease. Therefore, it verifies the need for the management and treatment of the affected individuals. Furthermore, having a definite diagnosis makes it easier for patients to accept the fact that they must live with such a serious condition.

A detected pathogenic mutation can help to identify affected family members with targeted screening for the known genetic variant. This method is quick and inexpensive. When the result is negative, the disease can be excluded and unnecessary follow-ups can be avoided, no further management is required. Ruling out the possibility of the disease has a great influence on people’s anxiety level and therefore the quality of life, as a significant difference was reported on the scores of trait anxiety between the Marfan and the normal population [46]. In the case of a positive test, adequate treatment and patient management need to be initiated. It is most useful in younger patients, as in most families phenotype only becomes apparent with increasing age [1], which makes the early diagnosis uncertain and delays the definite one. The clinical importance of early clinical diagnosis is the possibility of having a greater effect of early treatment to prevent the serious complications of the disease.

Patients can also benefit from knowing their disease-causing mutation in the process of family planning. The parents could make their decision of having children with the information on their risk of passing on the disease to their offspring. A heterozygous MFS patient and a healthy individual have a 50% chance of passing on the mutation to their child.

A dynamically evolving field, where the identified mutation could be useful is preimplantation genetic diagnostics [47].

Seven percent of the identified (likely) pathogenic variants affected some of the genes associated with LDS, which is a related disorder of MFS with a potentially more aggressive clinical presentation. Therefore, it requires a different therapeutic approach, including follow-up with extended imaging and a lower aortic diameter threshold for prophylactic aortic root replacement surgery [17]. Our patients with LDS had a significantly lower systemic score and they showed a tendency to be younger than people with MFS. However, they both resulted in severe CV manifestations. In Hungary, our research group was the first that carried out genetic testing for LDS patients. The significance of making the right diagnosis can be clearly seen, and due to the overlapping clinical features, in certain cases, it can be achieved by using gene-panel testing.

Furthermore, 63% (5/8) of VUS were found in genes other than *FBN1*.

Our finding that the mutations affecting the *FBN1* gene led to a significantly higher systemic score than the ones found in other genes, could indicate the need for gene panel testing even in patients with Marfan-like characteristics whose systemic score does not reach the threshold of 7 points.

The considerable number of non-*FBN1* mutations identified suggests that gene panel testing should be preferred instead of single gene screening in patients with the suspicion of Marfan syndrome and related disorders.

The clinically most relevant use of genotype–phenotype correlations is identifying genetic variants which are associated with more severe CV involvement.

We found that the combined group of HI and DN Cys mutations led to aortic involvement (aortic dissection, aortic dilation) significantly more frequently than DN non-Cys genetic variants. Furthermore, aortic surgery was significantly more common among patients with DN Cys mutations than in the other two groups. Our results show a strong correlation between the type of mutation and the severity of CV manifestations, which has the potential to improve the risk stratification of the patients in order to optimise the decision making on the necessity and timing of prophylactic aortic root surgeries.

Some articles focusing on the connections between the genotype and CV involvement have been published. Becerra-Muñoz and his colleagues carried out genetic testing for 108 individuals with suspected MFS and they detected 90 *FBN1* mutations. They found that patients with MFS and truncating variants had a higher percentage of aortic events than patients with a missense mutation [15]. Similarly, after examining 179 probands with pathogenic or likely pathogenic mutations, Baudhuin and colleagues reported a higher frequency of truncating and splicing variants in patients with an aortic event. They also found that patients with these mutations had an aortic event at a younger age than people with a missense variant [48]. The results of Franken et al. also support these findings by stating that HI patients have a 2.5-fold higher risk of CV death, a 2.4-fold increased risk of reaching the combined endpoint of dissection or death and a 1.6-fold higher risk of any aortic complication compared to DN mutations [49]. These articles treat the missense mutations as a homogenous group, and they all consider this mutation type as lower risk for serious CV manifestations. However, we further differentiated the DN group and we identified a subgroup that seems to be even more dangerous than the HI genetic variants.

Our findings support the results published by Takeda et al. on this topic. They also identified a subgroup within the DN mutations with a higher CV risk. As they reported, DN variants affecting or creating cysteine residues and in-frame deletions in exons 25–36 and 43–49 (DN-CD) had a 6.3-fold higher risk for aortic events than the other DN mutations (DN-non CD), which is comparable with or more deleterious than HI variants. The growth of the aortic diameter also appeared to be larger in the DN-CD + HI group than in the DN-non CD one [40]. Faivre et al. reported a significantly higher probability of ascending aortic dilation in patients with a mutation eliminating a cysteine than in patients with a variant resulting in cysteine creation [16]. These results also confirm the relevance of differentiating among DN mutations; however, we propose that DN Cys genetic variants could be considered higher CV risk than HI ones as they reached the need for aortic surgery significantly more frequently. There was no difference in their age at the last follow-up. More investigations need to be carried out to support this idea. Until then DN Cys and HI variants should be treated equally as high risk in the clinical practice. The location of the mutation can also have an impact on aortic involvement. In the above mentioned study by Faivre et al., mutations in exons 24–32 led to ascending aortic dilation significantly more frequently than variants found in other exons [16]. In contrast, our results did not show significant difference in terms of aortic involvement in patients with variants in exons 24–32 compared to individuals with mutations in other parts of the gene. However, the effect of the location of mutations is worth further investigating in future studies. The relevance of these studies is also highlighted by the finding that some patients with a DN non-Cys variant underwent aortic surgery at younger age than patients with DN Cys and HI mutations, even if no statistical difference could be observed in terms of age at the time of surgery among the mutation types. This could indicate that certain factors like location of mutation or affected domains may also influence CV severity. We are going to continue our study by involving further patients for gene panel testing. We also need to consider whether adding more genes to our panel would be a cost-effective way of increasing our detection rate.

## Limitations

We acknowledge the limitations of our study, including the small sample size. However, it is comparable to the sample sizes of previous studies on this topic and we were able to draw significant conclusions on the studied cohort.

We analysed a limited number of genes, therefore our multi-gene panel did not cover all the known disease-causing genes. In addition, the applied targeted screening method has its limitations, such as incomplete coverage and the need for regularly updating the gene panel due to novel gene-disease associations [10]. Despite these, our study successfully demonstrated the importance of using a multi-gene panel for patients with Marfanoid habitus.

Deep/non-canonical intronic regions were not sequenced, which could be a further limitation of our study [50].

Most of the patients in our study were started on antihypertensive drugs at the time of diagnosis of MFS or when significant and/or progressive aortic dilation was observed. Therefore, we cannot evaluate the contribution of the specific mutation and of the medication to the severity of aortic phenotype. However, as most MFS patients take blood pressure medications to prevent aortic complications, our study still provides relevant findings on the different effect of specific mutations on the severity of CV manifestations. It would be highly beneficial to carry out prospective studies to investigate the response of specific mutations to cardiac medications.

Prospective studies are needed to produce results that could be used in clinical practice in terms of genotype and CV manifestations. Despite the retrospective design of our study, it still provides significant findings on the correlations between genetic background and aortic involvement, and it could provide a base for further research.

## Conclusions

**Fig. 4:**
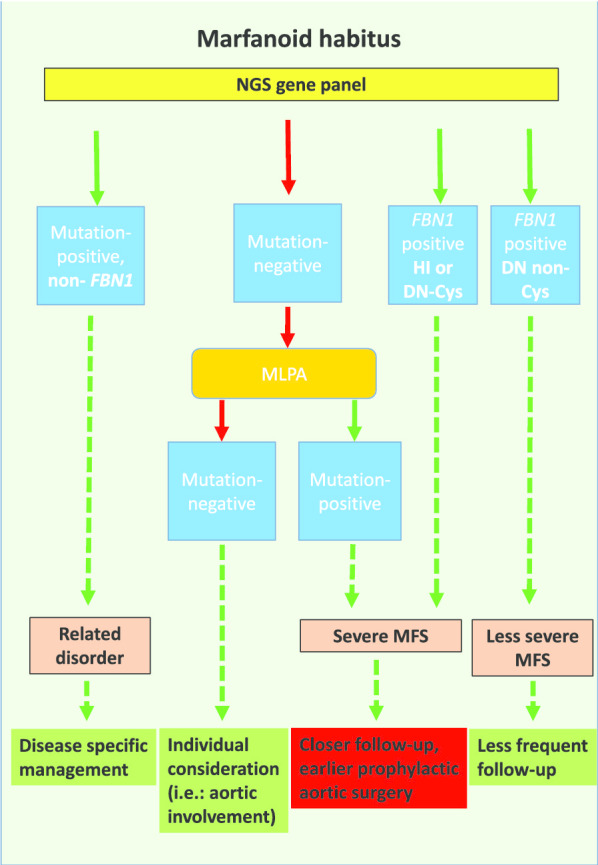
Recommended screening algorithm. People with suspected Marfan syndrome should undergo genetic screening. We recommend the use of a gene panel, followed by MLPA in negative cases. When HI or DN Cys mutations are identified, closer follow-up and earlier prophylactic surgery should be considered. DN non-Cys sequence variants should be managed as stated in the current ESC guidelines. When a (likely) pathogenic variant is detected in a gene other than the *FBN1*, then appropriate management of the identified disease/syndrome needs to be carried out. The management of people without a detected mutation should be based on the clinical presentation, mostly focusing on aortic involvement

Based on our results, we propose that the optimal way of genetic testing of MFS is the use of a gene panel, including *FBN1* and MFS-related genes, combined with CNV analysis with MLPA, if necessary. A summary of the recommended algorithm can be found in Fig. 4. This method can help to make the exact diagnosis and increase the detection rate.

We emphasize the need for genetic testing for patients who show Marfanoid features but their systemic score is below 7, as LDS patients may have lower scores, but they are likely to have severe CV manifestations.

MFS patients with DN Cys and HI mutations are at increased risk for aortic involvement, and DN Cys mutations seem to lead to the most severe aortic involvement. This finding could have an impact on patient management; however, further research is required to enable genetic information to be included in the risk stratification.

## Supplementary information

**Additional file 1:** Identified (likely) pathogenic mutations.

## Data Availability

The datasets used and/or analysed during the current study are available from the corresponding author on reasonable request.

## Abbreviations

*ACTA2*: Actin alpha 2, smooth muscle gene
CNV: Copy number variation
*COL3A1*: Collagen type III alpha 1 chain gene
CV: Cardiovascular
DN: Dominant negative
DN-CD: DN variant affecting or creating cysteine residues and in-frame deletion variants in the central cb-EGF domains (exons 25–36 and 43–49)
DN-Cys: DN mutation eliminating a disulphide-bonding cysteine
DN-nonCD: DN variant not affecting nor creating cysteine residues and in-frame deletion variants in the central cb-EGF domains (exons 25–36 and 43–49)
DN non-Cys: DN mutations not eliminating a cysteine
ESC: European Society of Cardiology
*FBN1*: Fibrillin-1 gene
*FBN2*: Fibrillin-2 gene
FTAAD: Familial thoracic aortic aneurysm and dissection
HI: Haploinsufficient
*KCNN1*: Potassium calcium-activated channel subfamily N member 1 gene
LDS: Loeys-Dietz syndrome
MFS: Marfan syndrome
MLPA: Multiplex ligation-dependent probe amplification
*MYH11*: Myosin heavy chain 11 gene
NGS: Next-generation sequencing
*SMAD3*: SMAD family member 3 gene
TGF-β: Transforming growth factor-β
*TGFB2*: Transforming growth factor beta 2 gene
*TGFB3*: Transforming growth factor beta 3 gene
*TGFBR1*: Transforming growth factor beta receptor 1 gene
*TGFBR2*: Transforming growth factor beta receptor 2 gene
vEDS: Vascular Ehlers-Danlos syndrome
VUS: Variants of unknown significance
