# Supplementary material for: Optimising the mutation screening strategy in Marfan syndrome and identifying genotypes with more severe aortic involvement
Source: Orphanet J Rare Dis. 2020 Oct 15;15:290. doi: 10.1186/s13023-020-01569-4 (PMC7558671; doi:10.1186/s13023-020-01569-4)
Supplement: Supplementary file 1 — Additional file 1: Identified (likely) pathogenic mutations. [file 13023_2020_1569_MOESM1_ESM.pdf]

Aortic involvement	Variant classification	Gene	Exon	Nucleotide change	Amino acid change	Effect of mutation	Mutation type
dilation	Likely Pathogenic	FBN1	50	c.6158G>A	p.Cys2053Tyr	missense	DN Cys
dilation	Likely Pathogenic	FBN1	32	c.3959G>C	p.Cys1320Ser	missense	DN Cys
dilation	Likely Pathogenic	FBN1	5	c.400T>C	p.Cys134Arg	missense	DN Cys
dilation	Likely Pathogenic	FBN1	34	c.4171T>C	p.Cys1391Arg	missense	DN Cys
dilation and dissection	Likely Pathogenic	FBN1	63	c.7769G>A	p.Cys2590Tyr	missense	DN Cys
dilation and dissection	Likely Pathogenic	FBN1	60	c.7375T>C	p.Cys2459Arg	missense	DN Cys
no	Likely Pathogenic	FBN1	34	c.4171T>C	p.Cys1391Arg	missense	DN Cys
dilation and dissection	Likely Pathogenic	FBN1	5	c.407G>A	p.Cys136Tyr	missense	DN Cys
dilation	Likely Pathogenic	FBN1	21	c.2462G>A	p.Cys821Tyr	missense	DN Cys
dilation	Likely Pathogenic	FBN1	54	c.6542G>T	p.Cys2181Phe	missense	DN Cys
dissection	Likely Pathogenic	FBN1	64	c.7897T>C	p.Cys2633Arg	missense	DN Cys
no	Likely Pathogenic	FBN1	64	c.7897T>C	p.Cys2633Arg	missense	DN Cys
dilation	Likely Pathogenic	FBN1	37	c.4472G>T	p.Cys1491Phe	missense	DN Cys
dilation	Likely Pathogenic	FBN1	44	c.5416T>G	p.Cys1806Gly	missense	DN Cys
dilation	Likely Pathogenic	FBN1	38	c.4691G>A	p.Cys1564Tyr	missense	DN Cys
dilation	Pathogenic	FBN1	58	c.7168T>C	p.Cys2390Arg	missense	DN Cys
dilation	Likely Pathogenic	FBN1	19	c.2287T>G	p.Cys763Gly	missense	DN Cys
dilation	Likely Pathogenic	FBN1	24	c.2809T>C	p.Cys937Arg	missense	DN Cys
no	Pathogenic	FBN1	25	c.2953G>A	p.Gly985Arg	missense	DN
dilation	Likely Pathogenic	FBN1	55	c.6697C>T	p.Pro2233Ser	missense	DN
no	Likely Pathogenic	FBN1	8	c.839A>T	p.Asn280Ile	missense	DN
no	Pathogenic	FBN1	2	c.32T>A	p.Leu11Gln	missense	DN
dissection	Likely Pathogenic	FBN1	17	c.2113G>A	p.Ala705Thr	missense	DN
dilation	Likely Pathogenic	FBN1	59	c.7208T>C	p.Ile2403Thr	missense	DN
dilation	Likely Pathogenic	FBN1	34	c.4210G>A	p.Asp1404Asn	missense	DN
dilation and dissection	Pathogenic	FBN1	62	c.7606G>A	p.Gly2536Arg	missense	DN
dilation	Likely Pathogenic	FBN1	53	c.6448C>T	p.Arg2150Cys	missense	DN
no	Pathogenic	FBN1	64	c.8038C>T	p.Arg2680Cys	missense	DN
no	Pathogenic	FBN1	16	c.1879C>T	p.Arg627Cys	missense	DN
no	Pathogenic	FBN1	2	c.32T>A	p.Leu11Gln	missense	DN
dilation	Pathogenic	FBN1	25	c.2988T>A	p.Cys996STOP	nonsense	HI
no	Pathogenic	FBN1	66	c.8326C>T	p.Arg2776STOP	nonsense	HI
dilation	Pathogenic	FBN1	59	c.7240C>T	p.Arg2414STOP	nonsense	HI
no	Pathogenic	FBN1	16	c.1912G>T	p.Glu638STOP	nonsense	HI
dilation	Pathogenic	FBN1	16	c.1912G>T	p.Glu638STOP	nonsense	HI
dilation	Pathogenic	FBN1	44	c.5368C>T	p.Arg1790STOP	nonsense	HI
dilation	Pathogenic	FBN1	44	c.5368C>T	p.Arg1790STOP	nonsense	HI
dilation	Pathogenic	FBN1	56	c.6856G>T	p.Gly2286Ter	nonsense	HI
dilation	Pathogenic	FBN1	29	c.3534T>G	p.Tyr1178Ter	nonsense	HI
dilation	Pathogenic	FBN1	10	c.1090C>T	p.Arg364Ter	nonsense	HI
dilation	Pathogenic	FBN1	10	c.1063A>T	p.Lys355Ter	nonsense	HI
dilation	Pathogenic	FBN1	38	c.4615C>T	p.Arg1539Ter	nonsense	HI
no	Pathogenic	FBN1	14	c.1693C>T	p.Arg565Ter	nonsense	HI
dilation	Pathogenic	FBN1	14	c.1693C>T	p.Arg565Ter	nonsense	HI
dilation	Pathogenic	FBN1	66	c.8326C>T	p.Arg2776Ter	nonsense	HI
dilation	Pathogenic	FBN1	46	c.5658Adel13ins8	p.Gln1886Valfs21*	frameshift	HI
dilation	Pathogenic	FBN1	8	c.762delC	p.Leu256Serfs*74	frameshift	HI
dilation	Pathogenic	FBN1	8	c.762delC	p.Leu256Serfs*74	frameshift	HI
dilation	Pathogenic	FBN1	66	c.8474delG	p.Gly2825Glufs*21	frameshift	HI
no	Pathogenic	FBN1	66	c.8474delG	p.Gly2825Glufs*21	frameshift	HI
dilation and dissection	Pathogenic	FBN1	19	c.2232_2235delGACC	p.Thr745IlefsTer26	frameshift	HI
dilation	Pathogenic	FBN1	7	c.648delC	p.Trp217GlyfsTer113	frameshift	HI
no	Pathogenic	FBN1	34	c.4140_4152del	p.Cys1380TrpfsTer29	frameshift	HI
dilation	Pathogenic	FBN1	39	c.4787_4796delGACCAAATCC	p.Arg1596LeufsTer41	frameshift	HI
dilation	Pathogenic	FBN1	24	c.2805delT	p.Gln936SerfsTer6	frameshift	HI
dilation	Pathogenic	FBN1	43	c.5244_5245delTG	p.Cys1748TrpfsTer18	frameshift	HI
dilation and dissection	Pathogenic	FBN1	16	c.1936_1951dupGGTCTGGATGGCCGTG	p.Val651GlyfsTer9	frameshift	HI
dilation	Pathogenic	FBN1	intron 38	c.4748-3T>G		splice	HI
dilation	Pathogenic	FBN1	intron48	c.5917+1G>A		splice	HI
dilation	Pathogenic	FBN1	intron45	c.5545+1G>T		splice	HI
dilation	Pathogenic	FBN1	intron 44	c.5423-2A>C		splice	HI
dilation	Pathogenic	FBN1	intron 35	c.4337-1G>T		splice	HI
dilation	Pathogenic	FBN1	intron 17	c.2114-1G>A		splice	HI
dilation	Pathogenic	FBN1	intron 46	c.5671+1G>A		splice	HI
dilation	Pathogenic	FBN1	intron 35	c.4337-2A>G		splice	HI
dissection	Pathogenic	FBN1	intron 51	c.6313+1G>T		splice	HI
dilation	Pathogenic	FBN1	intron 51	c.6313+1G>T		splice	HI
dilation	Pathogenic	FBN1	intron 51	c.6313+1G>T		splice	HI
dilation	Pathogenic	FBN1	intron 24	c.2855-2A>C		splice	HI
dilation	Pathogenic	FBN1	intron 18	c.2168-1G>A		splice	HI
dilation	Pathogenic	FBN1	intron 18	c.2168-1G>A		splice	HI
dilation	Likely Pathogenic	FBN1	30	c.3632_3634delTCT	p.Phe1211del	in frame del	HI
dilation	Likely Pathogenic	FBN1	30	c.3632_3634delTCT	p.Phe1211del	in frame del	HI
dilation	Likely Pathogenic	FBN1	57	c.6943_6945delACC	p.Thr2315del	in frame del	HI
dissection	Pathogenic	FBN1	exon 3-4			CNV	HI
dilation	Pathogenic	FBN1	exon 1-2			CNV	HI
dilation	Pathogenic	FBN1	exon 2-4			CNV	HI
dilation and dissection	Pathogenic	FBN1	exon 2-4			CNV	HI
dilation	Pathogenic	TGFB2	7	c.1039_1040delCT	p.Leu347ThrfsTer6	frameshift	HI
dilation	Pathogenic	TGFB2	7	c.1039_1040delCT	p.Leu347ThrfsTer6	frameshift	HI
dilation	Pathogenic	TGFBR2	7	c.1558C>T	p.Arg520Ter	nonsense	HI
dilation	Likely Pathogenic	TGFBR1	4	c.605C>T	p.Ala202Val	missense	DN
dilation	Likely Pathogenic	TFGBR1	4	c.797A>T	p.Asp266Val	missense	DN
dilation	Likely Pathogenic	SMAD3	2	c.238C>T	p.Arg80Trp	missense	DN
